# Eye-Movement-Assisted Time–Frequency EEG Decoding for Multimodal Robotic Arm Control

**DOI:** 10.3390/jemr19040074

**Published:** 2026-07-07

**Authors:** Xiangyang Sun, Wenjun Zhang, Jiahua Wu, Xingwei Xiong, Haixia Mei

**Affiliations:** 1 School of Electronics and Information, Changchun University, Changchun 130022, China; 19842866545@163.com (X.S.); 19729361528@163.com (W.Z.); 15948065320@163.com (J.W.); 15844072065@163.com (X.X.); 2 Key Laboratory of Intelligent Rehabilitation and Barrier-Free for the Disabled (Ministry of Education), Changchun University, Changchun 130022, China

**Keywords:** multimodal human–computer interaction, brain–computer interface, motor imagery, eye movement signals

## Abstract

Brain–computer interface (BCI) technology has shown potential for future rehabilitation-related and assistive control applications. Nevertheless, single-modality electroencephalography-based motor imagery (EEG-MI) signals are susceptible to interference, whereas existing algorithmic models suffer from limited classification accuracy and insufficient actionable control commands for interactive devices, thereby impeding their practical deployment. To tackle these limitations, this study presents a multimodal human–computer interaction control scheme that integrates eye-movement command encoding with EEG motor imagery decoding. Self-collected EEG-MI and eye-movement datasets were established to support the proposed multimodal control framework. In this framework, eye movements are not used merely as auxiliary inputs, but are encoded as discrete commands for start, stop, grasp, and release, thereby reducing the command burden of EEG-MI decoding. The EEG-TransNet model is enhanced by integrating a time–frequency feature branch and replacing the original convolutional encoder with an adaptive multi-branch EEG feature gating module, strengthening the representation and fusion of multi-domain features. The model yields average classification accuracies of 86.96% and 88.73% on the BCI IV-2a dataset and the self-collected EEG dataset, respectively. Four independent SVM binary classifiers are adopted to identify four eye movement patterns. The EEG and eye movement classification results are binary-encoded to generate hardware-compatible control commands. Robotic-arm grasping experiments with healthy trained participants showed an average task completion time of 17 s, and the repeated grasping success-rate results further provide preliminary evidence for the real-time feasibility of the multimodal control framework under controlled laboratory conditions.

## 1. Introduction

The core goal of human–computer interaction (HCI) technology is to establish an efficient, natural, and reliable communication channel between human intentions and external devices. With the development of sensors, intelligent algorithms, and wearable devices, HCI methods have gradually expanded from traditional explicit operations such as keyboards and mice to interaction paradigms driven by multi-source physiological signals, including voice, electromyography, eye movements, and electroencephalography [1,2,3]. For example, voice interaction methods based on the body-conducted sound channel in earphones can improve speech reconstruction quality in noisy environments [4], while gesture recognition frameworks based on complex surface electromyography signals can enable multiple gesture recognition and prosthetic hand control [5]. These studies indicate that physiological signals are becoming an important input source for the next generation of HCI systems, especially in scenarios where physical functions are limited or traditional interaction methods are unavailable or inconvenient to use.

Motivated by future rehabilitation-related and assistive control applications, brain–computer interfaces (BCIs) have garnered widespread attention because they can bypass peripheral nerves and muscles, directly decoding brain activity into control commands for external devices [6,7]. Among them, motor imagery (MI)-based BCIs do not rely on actual physical movement; users only need to imagine actions such as moving the left hand, right hand, both feet, or tongue, which induce identifiable EEG rhythm changes. Consequently, MI-BCIs have been widely investigated in neurorehabilitation, smart wheelchairs, and robotic arm control research [8]. In recent years, research on EEG-MI signal classification has progressed continuously, employing methods such as convolutional neural networks, spiking neural networks, graph convolution, transfer learning, multi-scale convolutions, common spatial pattern, and attention mechanisms to enhance the feature extraction and classification performance of motor imagery EEG signals [9,10,11,12,13,14,15,16,17]. These studies have effectively advanced the development of EEG-MI decoding models and demonstrated the feasibility of using motor imagery signals as a source of control commands.

However, constructing a practical control system based solely on EEG-MI signals still presents obvious limitations. First, EEG signals have low amplitude and a low signal-to-noise ratio, and are susceptible to interference from electrooculographic signals, electromyographic signals, environmental noise, and variations in individual states. Their spatiotemporal features also exhibit strong nonstationarity, resulting in insufficient stability of the model in practical control scenarios. Second, most current EEG-MI studies mainly focus on improving offline classification accuracy, whereas real interactive devices require not only continuous control commands such as directional movement, but also discrete state commands such as start, stop, grasp, and release. If all control functions rely on motor imagery signals, the number of classification categories and the difficulty of decoding will increase, and users may also experience mental fatigue after prolonged concentration. Third, in practical robotic arm control tasks, control commands should simultaneously satisfy accuracy, uniqueness, real-time performance, and interpretability, which are difficult to achieve using a single EEG-MI modality. Therefore, introducing more intuitive and stable auxiliary interaction signals while preserving the advantages of EEG-MI-based intention recognition is an important issue for improving the practicality of BCI systems.

Multimodal human–computer interaction provides a feasible approach to addressing the aforementioned problems. Compared with single-modality input, multimodal interaction can exploit the complementarity of different physiological signals in terms of information representation, interference resistance, and control granularity, thereby improving system robustness and usability [18,19,20]. Eye movement-based interaction is characterized by strong intuitiveness, fast triggering, and low learning cost for users, and has been widely applied in virtual reality, assistive input, medical rehabilitation, and embedded interaction systems [21,22]. Existing studies have investigated gaze fixation, blink triggering, active eye tracking, pressure sensor-based blink detection, pupil detection, and embedded real-time eye detection, verifying the feasibility of using eye movement signals as input for interaction commands [23,24,25,26,27]. However, when eye movement signals are used alone for long-term control, users are prone to visual fatigue. Moreover, eye movements are more suitable for explicit discrete triggering tasks rather than long-duration fine directional control. Therefore, eye movement signals and EEG-MI signals exhibit a natural complementarity in control tasks: EEG-MI is more suitable for expressing directional movement intentions, whereas eye movement signals are more suitable for clear state commands such as start, stop, grasp, and release.

Some studies have begun to explore the joint application of EEG and eye movement signals. For example, Kang et al. [28] combined resting-state EEG with eye-tracking data for the identification of children with autism spectrum disorder. He et al. [29] constructed a remote sensing object detection dataset that synchronously records EEG and eye-tracking data to investigate human visual search and object recognition processes. These studies demonstrate the research value of integrating EEG and eye movement signals. However, existing work has mainly focused on cognitive recognition, assisted disease diagnosis, or dataset construction, and has not yet sufficiently addressed multimodal control for practical execution devices. Particularly in robotic arm control scenarios, the key issue is not merely to concatenate the two types of signals, but to reasonably assign control roles according to the characteristics of different modalities: EEG-MI is responsible for continuous directional control, while eye movement signals are responsible for discrete state triggering, and the resulting commands are converted into hardware-recognizable control instructions through a stable encoding method.

Based on the issues described above, this study proposes an EEG motor imagery control method combined with eye movement signals, aimed at implementing multimodal human–computer interaction control for a robotic arm platform. Specifically, the public BCI Competition IV dataset 2a (BCI IV-2a) (https://bbci.de/competition/iv/download/ accessed on 20 April 2024) [30] and a self-collected EEG-MI dataset were used for motor imagery signal decoding, and eye movement signals were collected under four paradigms: fixation, eye closure, single blink, and double blink. To address EEG-MI signal classification, this study improved the EEG-TransNet model [31] by introducing a time–frequency feature branch alongside the original mean and variance pooling features, enhancing the model’s ability to represent EEG frequency-domain rhythm information. Additionally, an adaptive multi-branch EEG feature gating module was designed to adaptively weight and fuse features from different branches, thereby improving the model’s utilization of multi-source EEG features. For eye movement signals, a support vector machine (SVM) classifier was employed to recognize the four types of eye actions. The classification results of EEG-MI and eye movement signals were then encoded in binary form to generate control commands directly recognizable by the robotic arm system. Therefore, the main distinction of this work lies in the task-specific command role assignment, the hardware-compatible binary command encoding, and the specific improvements to EEG-TransNet, rather than in a general fusion of EEG and eye-movement signals.

The main contributions of this study are as follows:

(1) A command-level eye-movement-assisted EEG-MI control framework is proposed for robotic-arm operation. Motor imagery signals are used to control the robotic arm in the upward, downward, leftward, and rightward directions, while fixation, eye closure, single blink, and double blink are used for discrete commands such as start, stop, grasp, and release, respectively. This design clarifies the role assignment between EEG-MI-based directional control and eye-movement-based state triggering.
(2) An improved EEG-TransNet model is proposed. A time–frequency feature branch is introduced during the feature extraction stage, and adaptive multi-branch EEG feature gating is used to enhance the adaptive fusion capability among mean-pooled features, variance-pooled features, and time–frequency features, thereby improving the classification performance of EEG-MI signals.
(3) Self-collected EEG-MI and eye-movement signal datasets were constructed, and a robotic-arm control experimental platform was established. The classification results of EEG-MI and eye-movement signals were encoded into hardware-compatible binary commands for robotic-arm execution. The preliminary feasibility of the proposed method was then evaluated through small-scale laboratory-based robotic-arm grasping experiments involving different geometric objects.

## 2. Experiment

**Figure 1:**
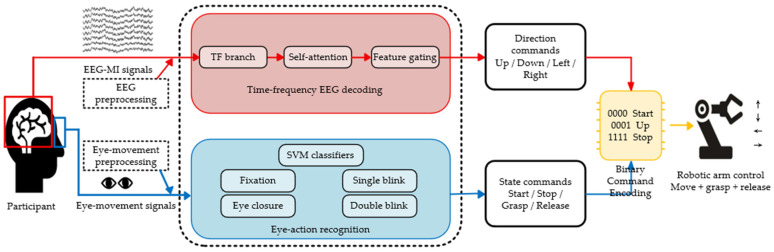
Flowchart of a multimodal robotic arm control system based on EEG-MI and Eye-Tracking signals.

The overall experimental workflow of the proposed EEG-MI and eye-movement-based multimodal robotic arm control system is shown in Figure 1. The workflow consists of two parallel signal-processing branches. In the EEG-MI branch, raw EEG-MI signals are acquired from participants and then preprocessed before being input into the improved EEG-TransNet model for feature extraction and classification. In the eye-movement branch, eye-movement signals are acquired and preprocessed, followed by feature extraction and SVM-based eye-action classification. The outputs of the two branches are further converted into binary commands and integrated through a priority-based collaborative control strategy to drive the robotic arm.

Specifically, in the EEG-MI branch, the raw EEG-MI data were prepared before model input by selecting the motor imagery time window from each trial and organizing each trial as a channel-by-time data matrix. Each trial was treated as one sample for subsequent model training and testing. Necessary signal quality checks were also performed before the data were input into the decoding model. The prepared EEG-MI samples were then fed into the improved EEG-TransNet model, in which the time–frequency branch, self-attention module, and adaptive multi-branch EEG feature gating module were used for feature extraction and classification to generate directional commands, including up, down, left, and right.

For the eye-movement branch, conventional preprocessing was performed to improve data quality and stability, including noise reduction, interpolation of missing data, and segment annotation. Eye-movement features were then extracted and classified using SVM classifiers to recognize fixation, eye closure, single blink, and double blink. These eye actions were further mapped to discrete state commands, including start, stop, grasp, and release. Finally, the EEG-MI and eye-movement classification outputs were encoded into hardware-recognizable binary commands to control the robotic arm.

### 2.1. Dataset

In this study, the public BCI Competition IV dataset 2a (BCI IV-2a) and a self-collected EEG motor imagery signal dataset were used as data sources for investigating EEG-MI signals. For eye movement signals, a self-collected dataset comprising four eye movement paradigms was adopted as the data source.

#### 2.1.1. BCI IV-2a Dataset

The BCI IV-2a dataset was constructed by the Brain-Computer Interface Laboratory at Graz University of Technology and contains EEG-MI data from nine healthy subjects. EEG signals were recorded using standard Ag/AgCl electrodes in a monopolar montage, with the reference electrode placed on the left mastoid and the ground electrode on the right mastoid. The sampling frequency was uniformly set to 250 Hz. The original recording montage contained 22 EEG channels and 3 EOG channels. In this study, the EEG channels were used as the main input for motor imagery decoding, and each EEG-MI trial was organized as a channel-by-time sample according to the selected motor imagery segment of the experimental paradigm. Each subject was required to perform four types of motor imagery tasks—left hand, right hand, both feet, and tongue—during the data acquisition experiments. Participants completed two acquisition sessions for training and evaluation on different days. Each session consisted of 288 trials, with 72 trials per class. The training session was used for model training, and the evaluation session was used only for testing. Therefore, the BCI IV-2a dataset followed a within-subject, session-separated training/testing protocol, with a 1:1 ratio between training and testing sets.

**Figure 2:**
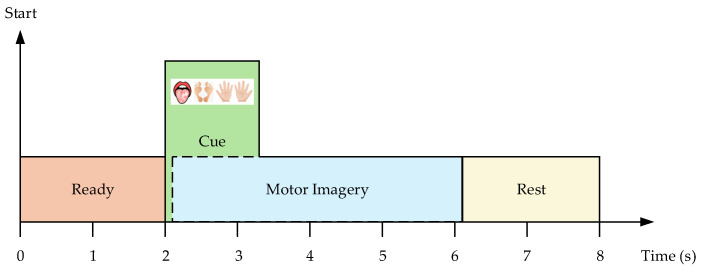
EEG-MI signal acquisition experimental paradigm diagram.

The experimental paradigm is illustrated in Figure 2. When the start cue appeared, the participant entered a preparation state. After 2 s, a cue icon corresponding to one of the motor imagery tasks appeared randomly on the screen and remained visible for 1.25 s. The participant was required to immediately imagine the indicated movement in their mind and continue this process until the end cue appeared. The motor imagery stage lasted for 4 s, during which no feedback was provided. After the task, the participant entered a 2-s relaxation state to prepare for the next start cue.

#### 2.1.2. Self-Collected EEG Motor Imagery Signal Dataset

The self-collected EEG-MI signal dataset in this study consists of EEG motor imagery data from six healthy participants. All participants signed informed consent forms before the formal experiment and were familiar with the data acquisition procedure. Before enrollment, all participants were confirmed to be physically and mentally healthy adults aged 20–30 years. Although a comprehensive clinical ophthalmological examination was not performed, the researchers conducted a basic pre-experiment confirmation with each participant. Participants were asked whether they had any obvious discomfort, whether they had any questions about the experimental procedure, and whether they were able to complete the required EEG-MI and eye-movement tasks. All participants confirmed that they could complete the experimental procedure and perform the required ocular actions, including fixation, eye closure, single blink, and double blink. The informed consent form also stated that appropriate rest intervals would be arranged during the experiment and that participants could request a break or withdraw from the experiment at any time if they felt uncomfortable. The acquisition device was an actiCAP slim/snap active-electrode EEG cap purchased from Brain Products GmbH, Germany, with supporting software including E-prime 3.0 and BrainVision Recorder 2. During data acquisition, 32 EEG channels were recorded and used to characterize the spatial-temporal patterns of motor imagery EEG signals. Before being input into the decoding model, each EEG-MI sample was represented as a channel-by-time matrix, where the channel dimension corresponded to the recorded EEG channels and the temporal dimension corresponded to the sampled time points within the selected motor imagery segment. The experimental paradigm was kept consistent with that of the BCI IV-2a dataset to ensure comparability between the two datasets. Each participant underwent five EEG data acquisition sessions. To prevent fatigue caused by continuous data collection, an interval of at least one day was maintained between any two sessions, ensuring that participants remained in good condition. The data acquisition process included four types of motor imagery tasks, with 20 sets of data collected per class per session. On-screen cues were presented randomly to ensure objectivity and accuracy. In total, 2400 EEG-MI samples were obtained from the six participants. The self-collected EEG-MI dataset was divided into training and testing sets using a stratified random sample-level split at an 8:2 ratio, so that the class distribution of the four motor imagery tasks remained consistent in both sets. The testing samples were excluded from model training, validation, and parameter selection.

For EEG-MI data, both the BCI IV-2a dataset and the self-collected EEG-MI dataset followed the same data-preparation procedure before model input. Specifically, the motor imagery time window was selected from each trial, and each EEG-MI trial was organized as a channel-by-time data matrix. Necessary signal-quality checks were performed before the data were input into the decoding model. The prepared EEG-MI trial matrices were then used as the input for all compared models.

#### 2.1.3. Self-Collected Eye Movement Signal Dataset

For eye movement signals, this study employed a self-collected dataset comprising four eye movement paradigms. The acquisition device was the Tobii Pro Fusion non-contact mobile eye tracker provided by Beijing Jinfa Technology Co., Ltd. (Beijing, China), with the acquisition frequency set to 120 Hz in the recording system. The dataset consisted of four paradigms: fixation, eye closure, single blink, and double blink. The experiment was organized in blocks and presented in an alternating manner, with appropriate rest intervals set between blocks. Each of the six participants provided 300 samples for each type of eye-movement signal, resulting in a total of 1800 samples per class. The four paradigms together formed a dataset containing 7200 eye-movement samples. The self-collected eye-movement dataset was divided into training and testing sets using a stratified random sample-level split at an 8:2 ratio. For the eye-movement data, the processing pipeline mainly included preprocessing, feature extraction, SVM-based classification, and command encoding. During preprocessing, denoising, interpolation of missing samples, and segment annotation were performed to improve signal quality and stability. For each eye-movement sample, features related to gaze trajectory, pupil diameter, eyelid opening/closure state, action duration, and blink-related temporal transitions were extracted and normalized before being used as the input of the one-vs-rest SVM classifier. The dataset was divided into training and testing sets while ensuring that the class distribution of fixation, eye closure, single blink, and double blink remained consistent in both sets. The testing samples were not used for SVM training or parameter selection. Therefore, the current eye-movement evaluation should be interpreted as a subject-pooled within-dataset evaluation rather than a subject-independent cross-subject validation. The classification outputs of the EEG-MI and eye-movement datasets were further encoded into robotic-arm control commands, as described in Section 2.2.1.

**Figure 3:**
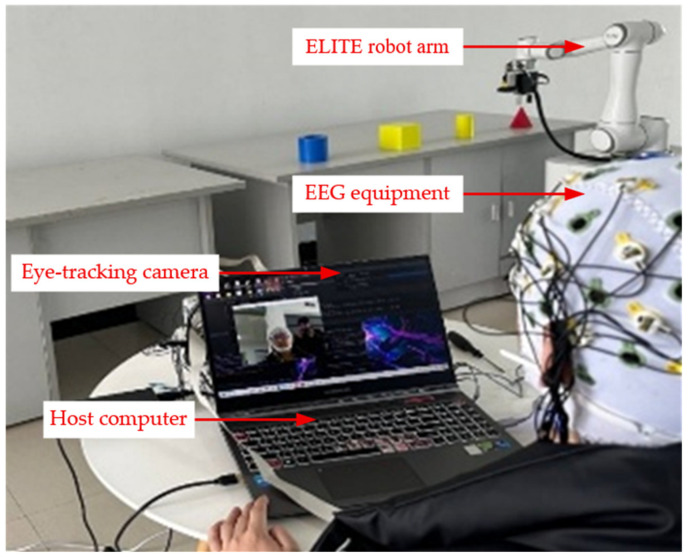
Hardware experimental platform diagram.

An eye movement-assisted EEG-MI hardware experimental platform was established. Data transmission was implemented via the TCP/IP protocol supported by the NB114 serial server. A local area network was configured to ensure that all devices were located within the same network segment. The remote host address was set to the host computer address (192.168.100.7), and the remote host port was set to the factory default port of the local device (8887). As shown in Figure 3, the experimental platform consisted of an ELITE Robot Arm, a Tobii Pro Fusion eye tracker, an EEG device, and a host computer. The robotic arm system contained predefined modifiable virtual coil regions. After the EEG motor imagery and eye movement signals acquired by the host computer were digitally converted into binary commands, virtual coils starting from M528 were set from 0 to 1 to execute different robotic arm actions. For example, after receiving the start command 0000, M528 was set to 1. When the upward movement command 0001 was subsequently received, M532 was set to 1. When both M528 and M532 were set to 1, the robotic arm system invoked the upward movement subroutine to complete the upward movement. When the end command 1111 was received, M529 was set to 1, and the program terminated. The functions corresponding to 1111-0000 and 1111-0000-1111-0000 were not related to the movement direction of the robotic arm, but instead triggered the robotic gripper Socket programs TOOLCLOSE and TOOLOPEN.

### 2.2. Model

#### 2.2.1. Eye-Movement Command Encoding and Priority-Based Collaborative Control Strategy

From the perspective of the control command reception mechanism, signals used to control the robotic arm should be efficient, accurate, unique, and stable. Digital commands are easier to process, transmit, and recognize in computing systems, making them particularly suitable for automated control scenarios. Therefore, this study adopts a digital encoding method for data transmission, with a binary format as the basic structure. The classification data of EEG motor imagery and eye actions are encoded in binary form to generate the corresponding binary commands for robotic arm movement.

**Figure 4:**
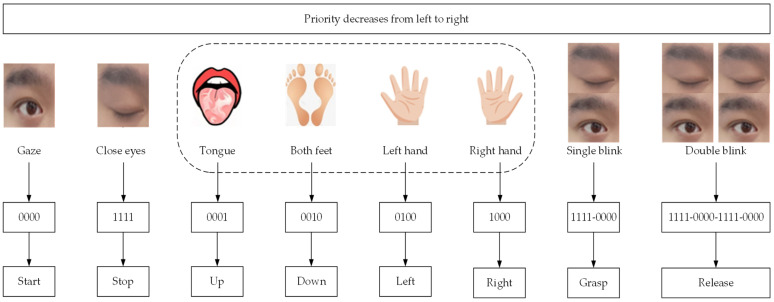
EEG motor imagery instruction encoding diagram combined with eye movements.

In this study, a binary encoding scheme for motor imagery signals assisted by eye movements was designed, as shown in Figure 4. The four-class EEG motor imagery results—tongue, both feet, left hand, and right hand—were mapped respectively to the upward, downward, leftward, and rightward movements of the robotic arm. Eye-movement signals were used as discrete operational commands rather than continuous motion commands, because EEG-MI signals are more suitable for directional movement control, whereas eye-movement signals are more suitable for explicit state triggering. In this scheme, the eye actions “fixation” and “eye closure” were used as the start and stop commands for robotic-arm movement, respectively, while “single blink” and “double blink” were used as the grasp and release commands, respectively.

The four ocular actions were selected according to their voluntary controllability, temporal distinguishability, and suitability for discrete command triggering. Fixation, eye closure, single blink, and double blink were assigned to the start, stop, grasp, and release commands, respectively. This design reduces the number of control commands that must be decoded from EEG-MI signals and improves the interpretability of the multimodal control strategy. To reduce the operational risk caused by accidental eye-command triggering, the command-priority strategy was further constrained by the task state. Before the start command was issued, the robotic arm did not execute EEG-MI directional movement commands. Once the system entered the control state, the stop command was assigned the highest priority. In addition, grasp and release commands were interpreted as discrete manipulation commands and were applied only in the corresponding manipulation stage. Eye actions that did not satisfy the predefined temporal rules were not converted into valid robotic-arm commands.

The priority assignment was designed according to the execution logic and safety requirements of the robotic-arm grasping task, as well as the functional division between the two modalities. The fixation and eye-closure commands are treated as system state-control commands. The start command activates the control system, whereas the stop command has the highest priority after system activation because it is used to interrupt the current control process when necessary. EEG-MI commands are responsible for directional movement and spatial positioning of the robotic arm. In contrast, the grasp and release commands are end-effector operations that are meaningful only after the robotic arm reaches the target position or enters the corresponding operation-ready stage. Therefore, grasp and release commands were assigned a lower priority than EEG-MI directional commands to avoid premature grasping, empty grasping, or interruption of the positioning process.

Command conflicts were handled using a state-based command parsing rule. In the idle state, the system only responds to the start command, while EEG-MI directional commands and grasp/release commands are ignored. After the system is activated, the stop command overrides all other commands. If a valid stop command is detected within the same control window, the system executes the stop operation and ignores other movement or end-effector commands. When no stop command is detected, EEG-MI directional commands are parsed first to complete the spatial positioning of the robotic arm. Grasp and release commands are accepted only when the robotic arm is in the corresponding operation-ready stage. If an EEG-MI directional command and a grasp/release command occur within the same control window, the directional command is executed first, whereas the grasp/release command is ignored in that window and needs to be triggered again after the robotic arm enters the appropriate operation stage. This rule avoids control ambiguity caused by simultaneous triggering of different command types.

#### 2.2.2. EEG-MI Signal Classification Model

**Figure 5:**
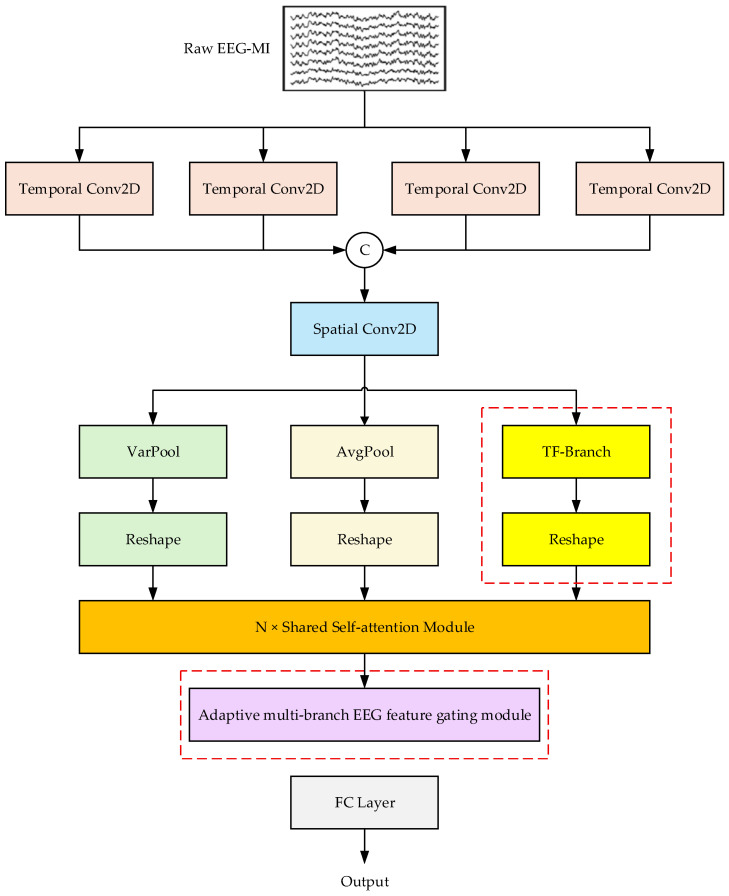
Overall architecture diagram of the improved EEG-TransNet model.

The overall architecture of the improved EEG-TransNet model in this study is shown in Figure 5. The model consists of four main components: a feature extraction module, a self-attention mechanism module, an adaptive multi-branch EEG feature gating module, and a classification module. Compared with the baseline EEG-TransNet model, the feature extraction module and the adaptive multi-branch EEG feature gating module are the key components improved in this study. These two modules are highlighted with red dashed boxes in Figure 5 and will be described in detail in Sections Feature Extraction Module and Adaptive Multi-Branch EEG Feature Gating Module.

For the self-attention mechanism module and the classification module, the same configuration as the original model is used. The self-attention mechanism module is designed to learn temporal dependencies among features from different time segments, enhancing the model’s decoding performance for EEG signals. This module mainly consists of two layers: the first layer is a multi-head attention (MHA) mechanism, which, compared with single-head attention, allows the model to simultaneously focus on information from different positions and subspaces, and the second layer is a fully connected feed-forward network (FFN), as shown in Equations (1) and (2). This network consists of two linear transformations with a GELU activation function, where erf(x) denotes the Gaussian error function. The computation process is repeated N times within the module, where N represents the depth of the self-attention mechanism module.

(1) $$GELU\left(x\right)=\frac{x}{2}\left(1+erf\left(\frac{x}{\sqrt{2}}\right)\right)$$

(2) $$FFN\left(x\right)=GELU\left(xW_{1}+b_{1}\right)W_{2}+b_{2}$$

After receiving the extracted features, the classification module uses a fully connected layer to perform classification. The predicted probabilities are then output through the softmax function, and the label with the highest probability is regarded as the final prediction.

To improve reproducibility, the main implementation settings of the EEG-MI decoding model are further specified here. The model was implemented in Python using PyTorch 2.0.1. Cross-entropy loss was used as the objective function for supervised training, and the Adam optimizer was adopted for parameter optimization. The feature dimension input to the self-attention module was set to dm = 32. The self-attention module contained four stacked self-attention layers, and each multi-head attention layer used eight attention heads; therefore, the dimension of each attention head was 4. The feed-forward network in the self-attention module used GELU as the activation function. The training parameters used in this study were learning rate = 0.001, epochs = 70, and batch size = 16. No dropout layer, additional L2 regularization, or weight-decay regularization was applied. No early stopping strategy was used; the training process was conducted with a fixed number of epochs, and the final model performance was evaluated on the testing set.

##### Feature Extraction Module

**Figure 6:**
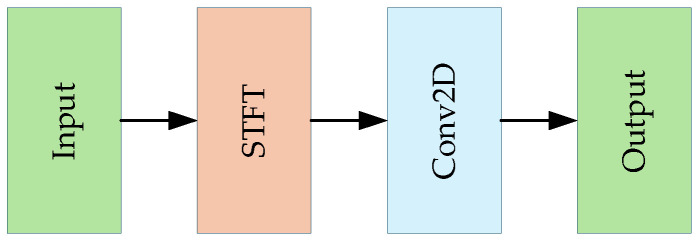
Structural diagram of the time–frequency feature branch.

The purpose of the feature extraction module is to capture meaningful features from EEG data. In our investigation, we found that most existing EEG-MI decoding models, including EEG-TransNet, rely heavily on single time-domain features and make insufficient use of frequency-domain information. To further improve the model’s decoding performance for EEG motor imagery signals, this study introduces a time–frequency feature branch in parallel with the mean pooling and variance pooling layers to enhance the model’s utilization of frequency-band information, as illustrated in Figure 6. EEG-MI signals are converted into time–frequency maps through short-time Fourier transform (STFT) and learnable filtering, and a two-dimensional convolutional layer is used to extract patterns of frequency-band energy variation over time, thereby supplementing frequency-band information. To further improve reproducibility, the detailed STFT settings used in the time–frequency feature branch are specified here. Each EEG-MI trial was represented using a 4-s motor imagery segment sampled at 250 Hz, resulting in 1000 sampling points per trial. STFT was applied to each EEG channel using a Hamming window with a window length of 128 samples, a hop length of 64 samples, an overlap of 64 samples, and an FFT length of 128. The center parameter was set to False to avoid additional boundary padding. Therefore, the frequency resolution was 1.95 Hz. Considering that motor imagery-related EEG rhythms are mainly distributed in the mu and beta bands, only the 8–30 Hz frequency range was retained for subsequent analysis. With these settings, the retained STFT representation had a size of 11 × 14 for each EEG channel. The magnitude-squared spectrum was further transformed into a log-power representation before being fed into the time–frequency branch.

After the STFT-based log-power representation was obtained, the learnable filtering and multi-branch feature extraction procedures were implemented as follows. The learnable filtering procedure was implemented using trainable convolutional kernels rather than fixed handcrafted filters, and these convolutional parameters were optimized jointly with the whole network during supervised training. For the retained EEG-TransNet backbone, the temporal filter bank consisted of four parallel two-dimensional convolutional branches with kernel sizes of (1,15), (1,25), (1,51), and (1,65), respectively. Each branch produced F1 = 8 feature maps. The outputs of the four branches were concatenated along the convolutional channel dimension, followed by batch normalization and ELU activation. A spatial convolutional layer with a kernel size of (*C*,1), where *C* denotes the number of EEG channels, was then used to learn inter-channel spatial dependencies.

The average-pooling and variance-pooling layers were applied along the temporal dimension with a kernel size of (1,50) and a stride of (1,15), respectively. In parallel, the time–frequency branch processed the STFT log-power representation with a size of *C* × 11 × 14 using one two-dimensional convolutional layer with a kernel size of (3,3), stride of 1, padding of 1, and 32 output channels. Batch normalization and ELU activation were applied after the convolutional operation. The frequency dimension was then aggregated by average pooling, and the temporal dimension was aligned with the mean-pooling and variance-pooling branches using linear interpolation. Finally, the time–frequency features were reshaped into a feature sequence with the same feature dimension as the other two branches, namely *dm* = 4 × *F*_1_ = 32. The aligned time–frequency features, mean-pooled features, and variance-pooled features were jointly fed into the shared self-attention module for subsequent feature fusion.

##### Adaptive Multi-Branch EEG Feature Gating Module

**Figure 7:**
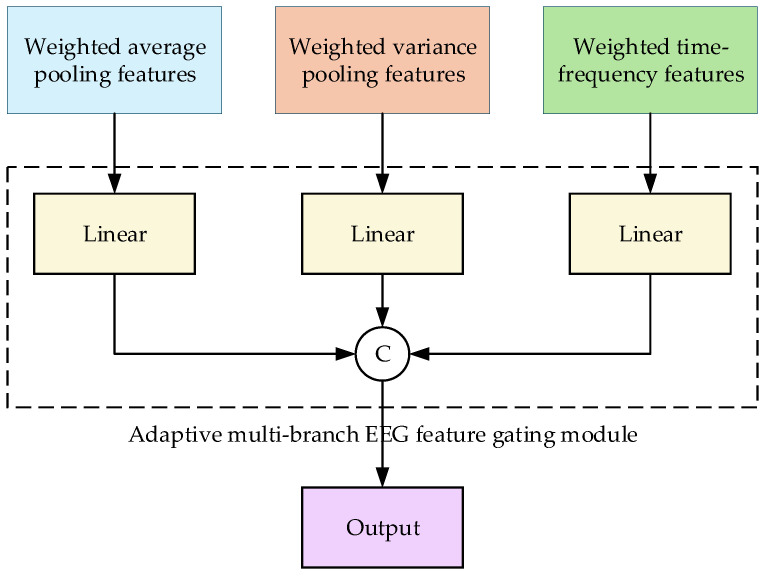
Structural diagram of the adaptive multi-branch EEG feature gating module.

In the original EEG-TransNet model, the weighted multi-branch EEG features output by the self-attention mechanism module are simply concatenated, lacking adaptive fusion. To address this, this study designed an adaptive multi-branch EEG feature gating module to replace the convolutional encoder, allowing better exploration of the relationships among mean-pooled features, variance-pooled features, and time–frequency features. The structure of the module is shown in Figure 7. Features weighted by the self-attention mechanism are fed into the gating mechanism, where a linear layer produces a “gating value,” as described in Equation (3). The gating mechanism assigns a weight to each feature, determining its contribution to the final output. Specifically, the linear layer maps feature *A* to a scalar representing its gating value *g_A_*, where *W_A_* is the weight matrix of the linear layer, *b_A_* is the bias term, and the sigmoid function ensures that the gating value lies within the range [0, 1].

(3) $$g_{A}=sigmoid\left(W_{A}A+b_{A}\right)$$

Compared with the convolutional encoder, which only processes local regional information, the adaptive multi-branch EEG feature gating module can better capture global temporal dependencies. The gating mechanism adaptively adjusts the weights of individual features for feature fusion, enabling the model to automatically modify its processing strategy according to the characteristics of the input data. This allows the model to more comprehensively understand the dynamic information of EEG signals, thereby improving decoding accuracy and stability.

#### 2.2.3. Eye Movement Signal Classification Model

In this study, eye actions were classified into four categories—fixation, eye closure, single blink, and double blink—according to the standard criteria designed in Table 1. The first two eye actions were identified based on the duration of the corresponding state. A state maintained in the same eye movement direction for more than 2 s was classified as “fixation.” An eye-closed state lasting for more than 1 s without reopening was classified as “eye closure.” The latter two eye actions were identified based on the number of eye-state changes within a 1.5-s window. Short eye-closure–opening transitions within this window were classified as blink events rather than sustained eye closure. One “eye closure–eye opening” state transition within 1.5 s was classified as a “single blink,” while two such state transitions were classified as a “double blink.”

The rules shown in Table 1 were used to define eye-action labels and extract temporal features. The same discrimination criteria and experimental instructions were applied to all participants to reduce ambiguity in eye-action labeling and improve the reproducibility of the four ocular command patterns across participants. For each eye-movement sample, the feature vector consisted of gaze trajectory features, pupil diameter features, eyelid opening/closure state features, blink-count features, and temporal duration features. Specifically, these features were used to describe gaze-point stability and displacement, pupil-size variation, eye opening and closure transitions, the number of blink events, and the duration of fixation, eye closure, or blink events. Before classification, all features were normalized to reduce the influence of scale differences among different feature dimensions. Based on the eye-action discrimination method, four one-vs-rest SVM classifiers with a radial basis function (RBF) kernel were trained to classify and predict the four eye-action categories. The one-vs-rest strategy transformed the *K*-class classification problem into *K* independent binary classification problems, as shown in Equation (4). Each binary classifier learns a decision function, where *w_i_* denotes the weight vector of the *i*-th classifier, $\phi \left(x\right)$ represents the feature mapping function, and *b_i_* is the bias term. For an input sample, the final class prediction is obtained using Equation (5).

(4) $$f_{i}\left(x\right)=w_{i}^{T}\phi \left(x\right)+b_{i}\;\;\;\;i=1,\ldots ,K$$

(5) $$\hat{y}=arg\underset{i=1,\ldots ,K}{max}f_{i}\left(x\right)$$

This study aimed to classify four types of eye actions: fixation, eye closure, single blink, and double blink. The eye-movement dataset was divided into training and testing sets using a stratified random split at an 8:2 ratio, ensuring that the class distribution of the four eye-action categories remained consistent in both sets. For each sample, features such as gaze trajectory stability, pupil diameter, eyelid opening/closure state, blink count, and temporal duration were extracted. Before classification, the extracted feature vectors were standardized using z-score normalization based only on the mean and standard deviation of the training set, and the same normalization parameters were applied to the testing set. Four binary SVM classifiers were trained using the one-vs-rest strategy, each corresponding to one eye-action category. The SVM classifiers used a radial basis function kernel. The penalty parameter C was selected from {0.1, 1, 10, 100}, and the kernel parameter gamma was selected from {0.001, 0.01, 0.1, 1}. Parameter selection was performed by five-fold cross-validation using only the training data. The testing samples were not used for SVM training, feature normalization parameter estimation, or hyperparameter selection. For each input eye-movement sample, the final predicted command was determined by selecting the class with the largest decision function value among the four binary classifiers. The same training, testing, and classification protocol was applied to the pooled samples from all participants.

## 3. Results and Discussion

### 3.1. Model Performance

#### 3.1.1. Training Details of the Improved EEG-TransNet Model

**Figure 8:**
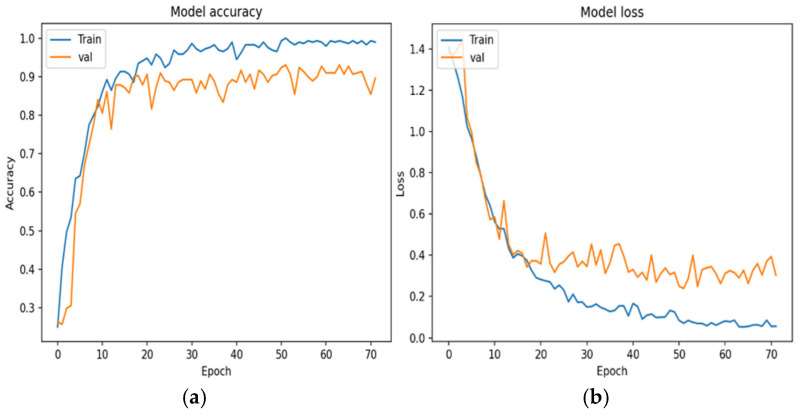
Effect of training epochs on the improved EEG-TransNet. (**a**) Line chart of model accuracy; (**b**) Line chart of model loss.

The environment configuration for the improved EEG-TransNet model was as follows: GPU: NVIDIA GeForce RTX 4060; programming language: Python 3.9.19; deep learning framework: PyTorch 2.0.1. Cross-entropy loss was used to monitor the convergence of the improved EEG-TransNet during training on the BCI IV-2a dataset. To observe the convergence trend, the training and validation curves were recorded over 100 epochs. As shown in Figure 8a, the accuracy on the training set increased rapidly and tended to stabilize after approximately the 30th epoch, while the accuracy on the validation set remained around 0.85 after the 50th epoch. In Figure 8b, the losses on both the training and validation sets decreased overall and no longer showed a significant drop after approximately the 50th epoch. Based on this convergence observation, the final training parameters used in the reported experiments were set as follows: learning rate = 0.001, epochs = 70, and batch size = 16.

Classification accuracy and the Kappa coefficient were adopted as the main evaluation metrics. In addition, to further evaluate the class-balanced recognition performance of the proposed model, precision, recall, and F1-score were calculated based on the confusion matrices of the test sets. For each class, precision, recall, and F1-score were defined as shown in Equations (6)–(9).

(6) $${Precision}_{i}=\frac{{TP}_{i}}{{TP}_{i}+{FP}_{i}}$$

(7) $${Recall}_{i}=\frac{{TP}_{i}}{{TP}_{i}+{FN}_{i}}$$

(8) $${F1}_{i}=\frac{2\times {Precision}_{i}\times {Recall}_{i}}{{Precision}_{i}+{Recall}_{i}}$$

(9) $$Macro\;F1=\frac{1}{N}\sum_{i=1}^{N}{F1}_{i}$$

where *TP_i_*, *FP_i_*, and *FN_i_* denote the number of true positives, false positives, and false negatives for the *i*-th class, respectively, and *N* denotes the number of classes. In this study, *N* = 4, corresponding to the four motor imagery classes. The Kappa coefficient is closely related to classification accuracy and the number of classes, as shown in Equation (10). A value closer to 1 indicates stronger classification and prediction capability of the model, where *p*_0_ denotes the average classification accuracy and *p_e_* denotes the chance agreement rate.

(10) $$Kappa=\frac{p_{0}-p_{e}}{1-p_{e}}$$

#### 3.1.2. Classification Accuracy of EEG-MI Signals Using the Improved EEG-TransNet Model

**Figure 9:**
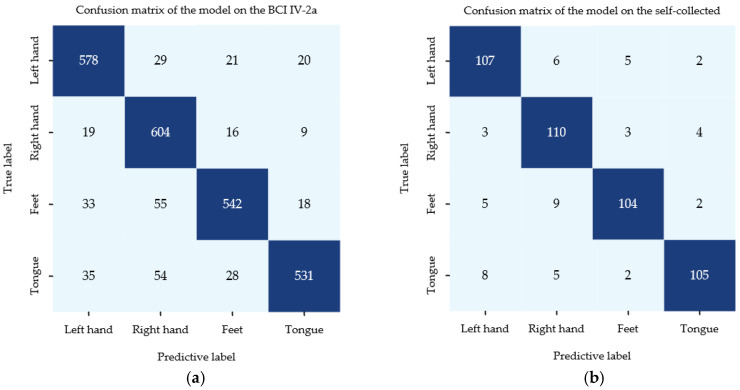
Confusion matrices of classification predictions by the improved EEG-TransNet on the test sets of the two datasets. (**a**) BCI IV-2a dataset; (**b**) Self-collected EEG signal dataset.

After training was completed, test data that had not participated in model training or parameter updating were input into the improved EEG-TransNet model to classify and predict the four motor imagery paradigms: left hand, right hand, both feet, and tongue. For the BCI IV-2a dataset, a within-subject session-separated training/testing protocol was used, where each subject’s training session was used for model training and the evaluation session was used only for final testing. The confusion matrix shown in Figure 9a was generated solely from the prediction results of the evaluation set. For the self-collected EEG-MI dataset, a stratified random sample-level split at an 8:2 ratio was used, and the testing samples were excluded from model training, validation, and parameter selection. The confusion matrix shown in Figure 9b was generated solely from the prediction results of the testing samples. The calculated average classification accuracies of the improved EEG-TransNet on the BCI IV-2a test set and the self-collected EEG-MI test set were 86.96% and 88.73%, respectively.

Based on the confusion matrices in Figure 9, the precision, recall, and F1-score of the proposed model were further calculated for each motor imagery class. On the BCI IV-2a dataset, the Macro-F1 of the proposed model was 86.99%. Specifically, the F1-scores for left hand, right hand, feet, and tongue were 88.04%, 86.91%, 86.37%, and 86.62%, respectively. On the self-collected EEG-MI dataset, the Macro-F1 was 88.77%, and the F1-scores for left hand, right hand, feet, and tongue were 88.07%, 88.00%, 88.89%, and 90.13%, respectively. These results are consistent with the accuracy and Kappa values and further indicate that the proposed model maintains relatively balanced recognition performance across different motor imagery classes.

To evaluate the reliability and stability of motor imagery signals in practical control, an EEG motor imagery classification experiment was conducted in combination with the hardware device. Four participants who had previously participated in EEG data acquisition and were familiar with the experimental procedure were invited to perform robotic arm directional control tasks under the same experimental conditions. Each participant conducted 100 trials for each of the four directional motor imagery control commands: upward, downward, leftward, and rightward. The number of correctly recognized commands and system response latency were recorded. The experimental results are shown in Table 2. The robotic arm achieved the highest recognition accuracy for the rightward movement at 89% and the lowest for the downward movement at 83.75%. The average recognition accuracy across the four movements was 86.63%, and the average system delay was only 0.1 s. The factors affecting the recognition accuracy of upward and downward movements may be attributed to individual differences in the clarity and stability of cortical activation during motor imagery tasks. Specifically, when imagining movements such as those of the feet or tongue, which are regulated by midline cortical areas, it is difficult to elicit stable features, resulting in reduced classification accuracy.

#### 3.1.3. Accuracy of SVM-Based Eye Movement Signal Classification

**Figure 10:**
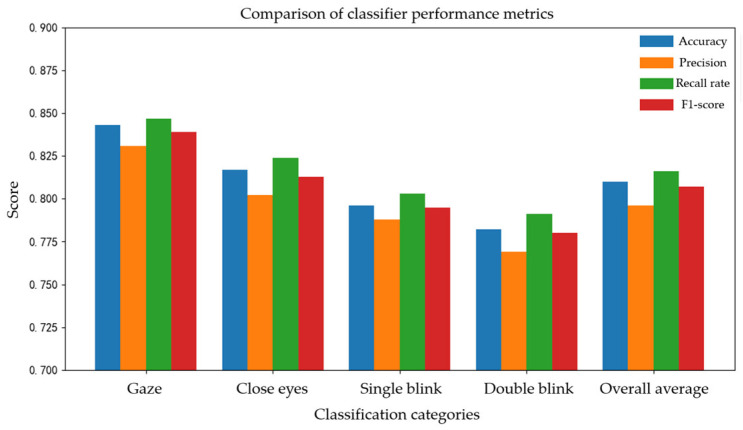
Bar chart of performance for the four eye movement classifiers.

In this study, four types of eye-movement signals were classified, and four binary classifiers were trained in parallel using the one-vs-rest SVM strategy. The performance of the eye-movement recognition module was evaluated on the testing samples that were not used for SVM training or parameter selection. The experimental results evaluating the classifier-level performance of the four eye-action commands are shown in Figure 10. As shown in Figure 10, the fixation classifier achieved the best performance, with an accuracy of 84.3%, precision of 83.1%, recall of 84.7%, and F1-score of 83.9%. The eye-closure classifier achieved an accuracy of 81.7%, precision of 80.2%, recall of 82.4%, and F1-score of 81.3%. The single-blink and double-blink classifiers showed relatively lower but comparable performance, with accuracies of 79.6% and 78.2%, respectively. Overall, the eye-movement recognition module achieved an accuracy of 81.0%, precision of 79.6%, recall of 81.6%, and F1-score of 80.7%.

In the robotic-arm control context, precision does not directly represent the standard false-positive rate; rather, it indicates the proportion of correctly recognized samples among those predicted as a specific eye-movement command. Therefore, lower precision may indicate a higher risk that a predicted command is incorrect. Recall indicates the proportion of correctly recognized samples among all samples belonging to a specific eye-movement command, and is therefore related to missed commands. The fixation classifier performed best because fixation has relatively stable eye-movement trajectories and duration-related characteristics. In contrast, the eye-closure, single-blink, and double-blink classifiers showed slightly lower performance, possibly because blink-related commands are short-duration ocular actions and are more easily affected by individual blinking habits, fatigue, blink amplitude, and incomplete eye closure. These results suggest that the selected ocular actions may show different levels of reproducibility across subjects. Nevertheless, all participants completed the four ocular action paradigms, and samples from multiple participants were used for classifier training and testing, indicating the preliminary feasibility of the proposed ocular command set for multimodal robotic-arm control. Therefore, the safety-related interpretation of eye-movement command recognition should consider both classifier-level performance and the command-priority strategy of the robotic-arm control system. In future work, subject-specific calibration and adaptive threshold adjustment will be considered to further improve cross-subject robustness.

#### 3.1.4. Real-Time Operation Verification

During the real-time robotic-arm grasping experiment, the above priority-based command parsing strategy was implemented in the control program and used throughout the complete task procedure, including system start, directional movement, grasping, releasing, and stopping. Therefore, the current experiment provides task-level feasibility evidence for the proposed priority strategy. However, it should be noted that this study did not conduct a separate command-conflict stress test covering all possible simultaneous-command scenarios. Accordingly, the experimental results should be interpreted as a preliminary feasibility validation of the priority-based control strategy in a laboratory grasping task rather than comprehensive evidence of its robustness in complex real-world scenarios.

**Figure 11:**
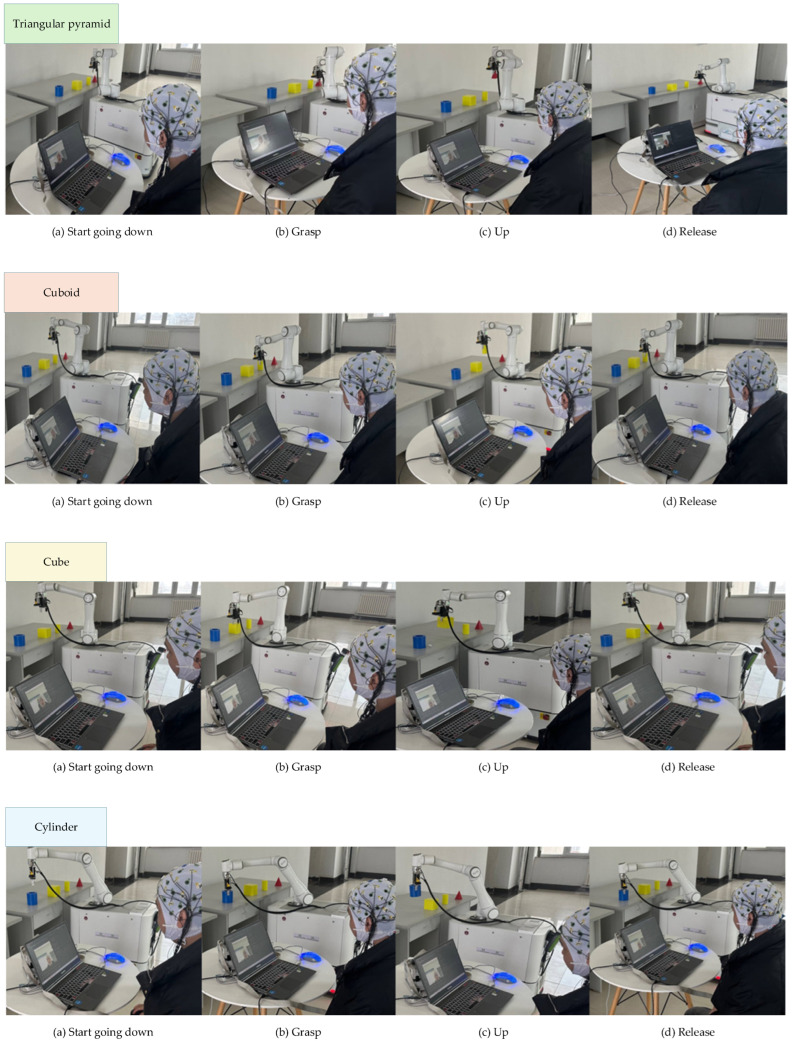
Representative scenes of the robotic-arm grasping experiment. The objects included a triangular pyramid, a cuboid, a cube, and a cylinder. The task procedure consisted of EEG-MI-based directional movement, eye-movement-triggered grasping, object transfer, and eye-movement-triggered release.

To preliminarily evaluate the feasibility of the experimental platform in a laboratory robotic-arm grasping task, this study conducted block-grasping experiments using a robotic arm. This experiment was designed as a small-scale proof-of-concept validation rather than a comprehensive usability or deployment-level evaluation. Four types of objects—triangular pyramid, cuboid, cube, and cylinder—were placed on the experimental table. Participants controlled the robotic arm through motor imagery and combined eye movement commands to perform grasping and placement. The participants were four individuals who had previously participated in the data acquisition experiments and were familiar with the operation procedures. Each participant performed 20 grasping trials for each type of geometric object. A complete grasping process consisted of the robotic arm moving from the initial position leftward or rightward to the position above the object, moving downward, grasping the object, lifting it upward, and placing it at the target location. Partial experimental scenes are illustrated in Figure 11.

The complete time taken by the four participants to perform each grasping task was recorded, and the mean of all timing data was calculated, as shown in Table 3. For example, the time recorded for Participant 1 to grasp the triangular pyramid represents the average completion time obtained from 20 trials of triangular pyramid grasping performed by Participant 1, which was 19 s.

**Figure 12:**
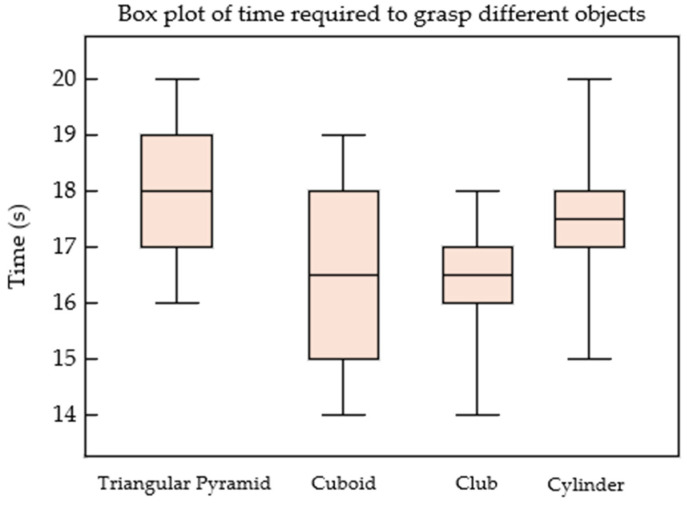
Box plot of the time required to grasp different objects.

A box plot was used to illustrate the distribution of the time required by the participants to grasp different geometric objects, as shown in Figure 12. In the task of grasping the triangular pyramid, the minimum and maximum completion times were 16 s and 20 s, respectively, corresponding to the lower and upper endpoints of the box plot. According to the lower and upper boundaries of the box, the first quartile (Q1) was 17 and the third quartile (Q3) was 19, indicating that 75% of the data were less than or equal to 19 s. This suggests that the grasping task could generally be completed within 17 to 19 s. Due to the pointed top of the triangular pyramid, participants required more time to grasp this type of object. In contrast, the other geometric objects were relatively easier to grasp, resulting in shorter average completion times.

#### 3.1.5. Success-Rate Evaluation of Repeated Robotic-Arm Grasping Tasks

To further evaluate the reliability of the proposed multimodal robotic-arm control framework, a repeated grasping-success experiment was conducted in addition to the completion-time evaluation. Different from the completion-time experiment, which mainly focused on task execution efficiency, the repeated grasping-success experiment was designed to assess whether the participants could stably complete the robotic-arm grasping task over multiple trials. Four participants who had been familiar with the experimental procedure and control strategy were invited to complete the experiment. Each participant performed 50 trials for each of the four object-grasping tasks, including triangular pyramid, cuboid, cube, and cylinder grasping. Therefore, each object type included 200 trials in total, and 800 repeated grasping trials were conducted across the four object types. The number of successful and failed trials was recorded, and the task success rate was calculated as the ratio of successful trials to the total number of trials. The results are shown in Table 4.

A successful trial was defined as a trial in which the participant continuously completed the full control procedure, including system activation, EEG-MI-based directional movement, target approach, grasping, object transfer, release, and task termination, and the target object was correctly grasped and placed at the specified position. A failed trial was defined as a trial in which the object was not successfully grasped, the object dropped during transfer, the release position was clearly incorrect, or the current trial had to be restarted due to continuous command errors. Command errors mainly included incorrect EEG-MI directional commands and false triggering or missed triggering of eye-movement-based grasp/release commands. For minor directional deviations, the participant was allowed to continue using EEG-MI directional commands to correct the robotic-arm position before triggering the grasp or release command. For errors that interrupted the task flow, the participant used the stop command to terminate the current process, and the trial was then restarted from the initial state.

The results show that the cuboid grasping task achieved the highest average success rate, whereas the triangular pyramid task showed the lowest average success rate. The lower success rate for the triangular pyramid may be related to the mismatch between the object geometry and the robotic gripper, which reduced contact stability during grasping. These success-rate results, together with the completion-time results, provide a more complete task-level evaluation of the proposed multimodal robotic-arm control framework. However, considering the limited number of participants and the laboratory setting, these results should be interpreted as preliminary task-level feasibility evidence rather than comprehensive proof of reliable practical deployment.

### 3.2. Comparison of Model Classification and Prediction Accuracy

To ensure a fair comparison, all compared models were trained and tested under the same experimental protocol. The same datasets, train/test splits, EEG-MI data-preparation procedure, validation strategy, and evaluation metrics were used throughout the comparative experiments. For the BCI IV-2a dataset, a within-subject protocol was adopted, in which the training session of each subject was used for model training and the evaluation session was used only for final testing. For the self-collected EEG-MI dataset, the same 8:2 training/testing split was applied, and the testing set was not involved in training, validation, or parameter selection. Accuracy and the Kappa coefficient were used as the common evaluation metrics.

To verify the performance of the deep learning model proposed in this study, comparative experiments on classification prediction accuracy were conducted with three model networks, namely MSHANet [32], TCNet-Fusion [33], and EEG-TransNet, using the BCI IV-2a and self-collected EEG motor imagery datasets. The experimental results of each model on the BCI IV-2a dataset are shown in Table 5, where bold values indicate the best results. As shown in Table 5, the improved EEG-TransNet model proposed in this study achieved the best decoding performance among the compared models in terms of both average classification accuracy and Kappa coefficient. The proposed model improved the average classification accuracy by 7.07, 3.34, and 1.75 percentage points compared with MSHANet, TCNet-Fusion, and EEG-TransNet, respectively. Compared with the selected baseline methods, the proposed model further incorporates a time–frequency feature branch and adaptive multi-branch EEG feature gating, which may enhance the utilization of complementary EEG features and improve decoding performance.

The self-collected EEG motor imagery dataset was applied to four deep learning models, with all networks retaining their original designs during the experiments. The results are shown in Table 6, where bold values indicate the best results. Certain differences in motor imagery classification accuracy were observed among different models across the subjects (S01–S06). Overall, the improved EEG-TransNet outperformed the other models, achieving an average classification accuracy of 88.73%. Owing to its ability to learn multi-scale time–frequency features, the proposed model improved the average classification accuracy by 5.98, 5.82, and 2.52 percentage points compared with MSHANet, TCNet-Fusion, and EEG-TransNet, respectively.

It should be noted that Table 5 and Table 6 retain accuracy and Kappa as the main cross-model comparison metrics, because these two metrics were uniformly obtained for all compared models under the same experimental protocol. The additional F1-score analysis was conducted for the proposed model based on the available test-set confusion matrices, aiming to provide supplementary evidence of class-balanced performance. Since AUC calculation requires continuous prediction scores or class probability outputs, and cannot be rigorously derived from confusion matrices alone, AUC values were not estimated in this revision to avoid reporting potentially misleading results.

### 3.3. Ablation Study

To further analyze the individual and combined effects of the proposed time–frequency branch and adaptive multi-branch EEG feature gating module, the ablation study was reorganized into a 2 × 2 module combination framework. In this framework, the original EEG-TransNet was regarded as the baseline model without the time–frequency branch and with the original convolutional encoder. The model without the time–frequency branch retained the proposed gating module, whereas the model without the gating module retained the time–frequency branch and used the original convolutional encoder. The complete proposed model contained both the time–frequency branch and the adaptive multi-branch EEG feature gating module. This design allows the effects of the two modified modules and their combination to be compared more clearly. The experimental results are shown in Table 7. In Table 7, the complete proposed model achieved the best overall performance on both datasets. Compared with the original EEG-TransNet, the proposed model improved the average accuracy from 85.21% to 86.96% on the BCI IV-2a dataset and from 86.21% to 88.73% on the self-collected EEG-MI dataset. When only the proposed gating module was retained without the time–frequency branch, the accuracies were 85.02% and 84.21% on the two datasets, respectively. When only the time–frequency branch was retained with the original convolutional encoder, the accuracies were 82.33% and 83.01%, respectively. These results indicate that using either module alone does not always lead to sufficient improvement. In contrast, the best performance was obtained when the time–frequency branch and the adaptive multi-branch EEG feature gating module were used together, suggesting that the two modules are complementary. The time–frequency branch enriches the representation of rhythm-related EEG-MI features, whereas the gating module adaptively fuses multi-branch features. Their joint use enables more effective multi-domain feature representation and fusion.

**Figure 13:**
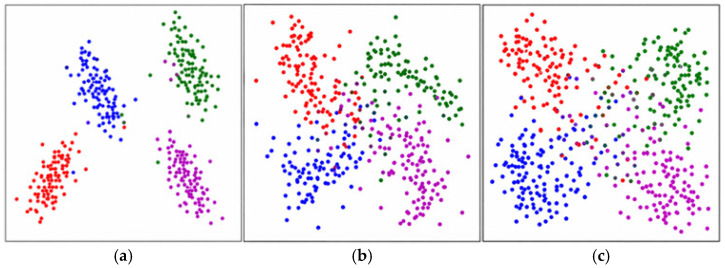
Feature classification visualization. (**a**) Improved EEG-TransNet; (**b**) Removal of the adaptive multi-branch EEG feature gating module; (**c**) Removal of the time–frequency feature branch.

Data from Subject 9 in the BCI IV-2a dataset were selected, and the t-SNE method [34] was used to visualize the learned features. The visualization results of the improved EEG-TransNet model, the model without the adaptive multi-branch EEG feature gating module, and the model without the time–frequency feature branch are shown in Figure 13, where different colors represent different classes. As shown in Figure 13a, the improved EEG-TransNet model produced clearly separated and compactly distributed feature clusters. In Figure 13b, when the adaptive multi-branch EEG feature gating module was removed, the feature distribution was similar to that in Figure 13a, but the boundaries between different classes became less distinct, making confusion more likely. In Figure 13c, when the time–frequency feature branch was removed, the classified features became relatively more dispersed.

### 3.4. Effectiveness of the Time–Frequency Feature Branch

**Figure 14:**
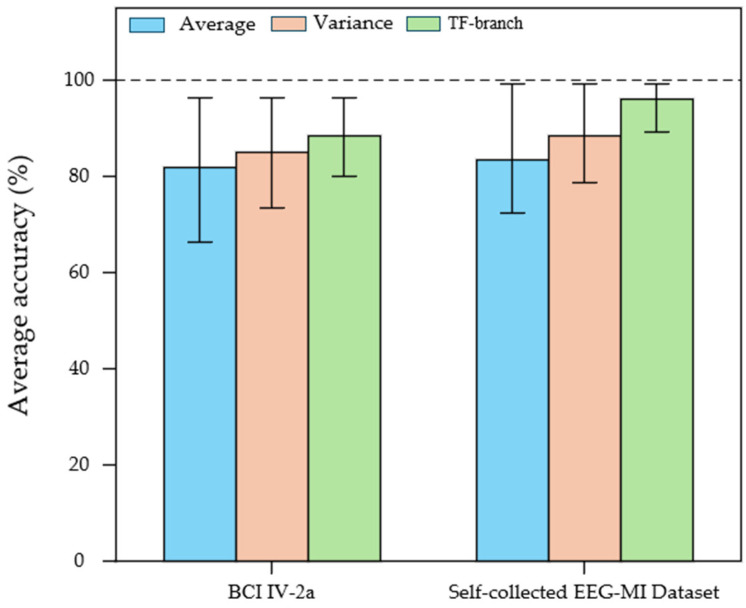
Descriptive comparison of average classification accuracy among the mean-pooling branch, variance-pooling branch, and time–frequency branch.

To verify the contribution of the time–frequency branch, the time–frequency branch, mean pooling, and variance pooling were separately used in the improved EEG-TransNet model to conduct classification accuracy experiments on the BCI IV-2a and self-collected EEG-MI datasets. The experimental results are shown in Figure 14. In the revised manuscript, this comparison is presented as a descriptive analysis of average classification accuracy among different feature branches. Compared with the mean-pooling and variance-pooling branches, the time–frequency branch showed higher average accuracy on both datasets, suggesting that the time–frequency representation may provide complementary rhythm-related information for EEG-MI decoding. Since the current comparison is intended to illustrate the average performance trend rather than to claim inferential statistical significance, the significance markers used in the original version have been removed.

### 3.5. Visualization of Learned Features

**Figure 15:**
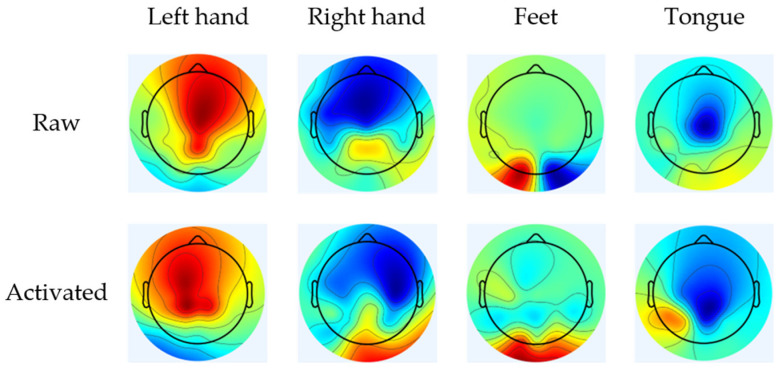
EEG topographic map on the BCI IV-2a dataset.

**Figure 16:**
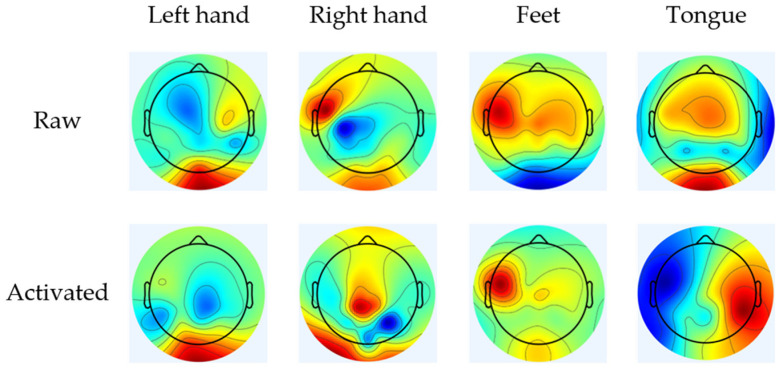
EEG topographic map on the self-collected EEG dataset.

To analyze the learned features of the improved EEG-TransNet, this study employed the Gradient-weighted Class Activation Mapping (Grad-CAM) method to activate EEG signals based on the learned features. In Grad-CAM [35], the gradients of each feature map are globally averaged to obtain the corresponding importance weights of the feature maps. These weights are then used to perform a weighted summation of the corresponding feature maps, producing an initial coarse heatmap. By resampling the EEG signals to their original scale on the coarse heatmap, the final heatmap is obtained. Finally, the original signals are multiplied by the final heatmap to generate activated signals, which are then visualized. In this study, EEG topographic maps of the original and activated signals for the BCI IV-2a and self-collected EEG motor imagery datasets are shown in Figure 15 and Figure 16. As can be seen from the figures, most of the original and activated signals are concentrated in the motor cortex region, which reflects the brain activity of the subjects during the corresponding MI tasks and indicates that the improved EEG-TransNet model can effectively extract meaningful features.

## 4. Conclusions

This study investigates the limitations of single-modal brain–computer interfaces in future rehabilitation-related and assistive control applications, including weak anti-interference ability, limited classification accuracy, and insufficient control instructions. A multimodal robotic arm control framework based on time–frequency EEG decoding and eye-movement command encoding is presented. By constructing self-collected EEG-MI and eye-movement datasets, a collaborative control paradigm is formulated in which EEG motor imagery is responsible for continuous directional control and eye movements are responsible for discrete command triggering, thereby enabling command-level complementary utilization of multimodal physiological signals. For EEG decoding, an enhanced EEG-TransNet model is developed by incorporating a time–frequency feature branch and employing an adaptive multi-branch EEG feature gating module, which strengthens the representation and adaptive fusion of multi-domain features. Experimental results demonstrate that the proposed model yields an average classification accuracy of 86.96% on the BCI IV-2a dataset and 88.73% on the self-collected EEG dataset, surpassing baseline models in decoding performance. For eye-movement recognition, the SVM classifier attains stable performance, with an average accuracy of around 80% for single and double blinks. By binary encoding, the system generates hardware-executable commands. The average recognition accuracy for robotic arm control reaches 86.63%, the average system latency is merely 0.1 s, and the average grasping task completion time is 17 s, providing preliminary evidence for the real-time feasibility of the proposed approach under controlled laboratory conditions. The proposed command-level multimodal collaborative control strategy may help improve the reliability and practicality of EEG-based robotic-arm control under controlled laboratory conditions. By combining EEG-MI directional intention decoding with eye-movement command triggering, the proposed method may provide a technical reference for future rehabilitation-robot, intelligent assistive-device, and barrier-free manipulation research.

However, the present study should be interpreted as a preliminary proof-of-concept validation under controlled laboratory conditions, rather than as clinical validation. The self-collected EEG-MI and eye-movement datasets involved six healthy young participants, and the real-time robotic-arm experiment involved four trained participants. Therefore, the current results mainly demonstrate the feasibility of the proposed multimodal control framework in healthy trained volunteers, rather than broad-population usability, direct clinical rehabilitation applicability, or verified rehabilitation outcomes. In addition, patients with motor impairment, neurological diseases, visual or oculomotor abnormalities, severe fatigue, or reduced cognitive endurance were not included. No comprehensive clinical ophthalmological examination was performed before enrollment, and factors such as ocular fatigue, individual blinking habits, head posture changes, illumination conditions, calibration errors, glasses reflection, or partial ocular occlusion may affect eye-movement command recognition in practical environments. The self-collected datasets were also evaluated using stratified random sample-level splits, which does not fully assess subject-independent or session-independent generalization.

Future studies will include larger and more diverse participant groups, patient populations, standardized ophthalmological screening, leave-one-subject-out and cross-session validation, cross-day repeated testing, independent external datasets, longer-term repeated-use evaluation, and more realistic rehabilitation-related scenarios. Future work will also consider subject-specific calibration, adaptive threshold adjustment, ocular fatigue monitoring, and more detailed command-level safety analysis, including confusion patterns, false-positive and false-negative rates, and command-conflict tests under more complex simultaneous-command conditions.

## Figures and Tables

**Table 1:** Eye movement signal discrimination method table.

Eye Movement Signals	Discrimination Method
Fixation	Lasting for more than 2 s
Eye closure	Lasting for more than 1 s
Single blink	One “eye closure–eye opening” event within 1.5 s
Double blink	Two consecutive “eye closure–eye opening” events within 1.5 s

**Table 2:** EEG motor imagery control hardware device action data table.

Participant	Number of Upward Movements	Number of Downward Movements	Number of Leftward Movements	Number of Rightward Movements	Delay
1	89	84	90	92	0.09 s
2	86	86	88	90	0.10 s
3	82	82	87	88	0.12 s
4	87	83	86	86	0.10 s
Average	86.00	83.75	87.75	89.00	0.10 s

**Table 3:** Completion time table for grasping different geometric objects in the experiment.

Participant	Time Required to Grasp the Triangular Pyramid	Time Required to Grasp the Cuboid	Time Required to Grasp the Cube	Time Required to Grasp the Cylinder	Overall Time Required to Grasp the Four Objects
1	19 s	15 s	18 s	15 s	16.75 s
2	16 s	19 s	16 s	17 s	17 s
3	17 s	14 s	14 s	20 s	16.25 s
4	20 s	18 s	17 s	18 s	18.25 s

**Table 4:** Success-rate results of the repeated robotic-arm object-grasping experiment.

Object Type	Total Trials	Successful Trials	Failed Trials	Average Success Rate	Maximum Success Rate	Minimum Success Rate
Triangular	200	140	60	70%	80%	62%
Cuboid	200	172	28	86%	94%	78%
Cube	200	164	36	82%	90%	74%
Cylinder	200	160	40	80%	86%	74%

**Table 5:** Classification accuracy of different models on the BCI IV-2a dataset.

Subject	MSHANet	Kappa	TCNet-Fusion	Kappa	EEG-TransNet	Kappa	Our Method	Kappa
	Accuracy (%)		Accuracy (%)		Accuracy (%)		Accuracy (%)	
A01	81.78	0.757	**90.58**	**0.874**	88.72	0.850	90.48	0.873
A02	68.15	0.575	70.52	0.607	65.18	0.536	**78.36**	**0.712**
A03	91.86	0.891	95.17	0.936	**96.40**	**0.952**	95.62	0.942
A04	75.47	0.673	76.64	0.689	85.31	0.804	**87.51**	**0.833**
A05	76.55	0.687	82.12	0.762	82.85	0.771	**87.67**	**0.836**
A06	67.21	0.563	68.70	0.583	73.45	0.646	**74.29**	**0.657**
A07	88.35	0.845	94.11	0.921	**95.32**	**0.938**	84.27	0.790
A08	83.49	0.780	88.79	0.851	90.15	0.869	**91.13**	**0.882**
A09	86.06	0.814	85.87	0.812	89.51	0.860	**93.21**	**0.910**
Average	79.89	0.732	83.62	0.781	85.21	0.803	**86.96**	**0.826**

**Table 6:** Classification accuracy of different models on the self-collected EEG dataset.

Subject	MSHANet	Kappa	TCNet-Fusion	Kappa	EEG-TransNet	Kappa	Our Method	Kappa
	Accuracy (%)		Accuracy (%)		Accuracy (%)		Accuracy (%)	
S01	77.69	0.703	76.22	0.682	78.12	0.708	**86.32**	**0.817**
S02	82.35	0.765	74.51	0.660	82.55	0.767	**84.14**	**0.789**
S03	79.74	0.730	85.24	0.803	83.88	0.785	**85.79**	**0.810**
S04	89.30	0.857	85.92	0.812	**92.80**	**0.904**	90.95	0.879
S05	86.63	0.821	90.58	0.874	91.30	0.884	**91.56**	**0.887**
S06	80.78	0.743	84.99	0.800	88.61	0.848	**93.62**	**0.915**
Average	82.75	0.770	82.91	0.772	86.21	0.816	**88.73**	**0.849**

**Table 7:** A 2 × 2 ablation analysis of the proposed EEG-TransNet.

Dataset	Variant	TF	Fusion	Acc. (%)	Kappa
BCI IV-2a	Baseline	×	Conv.	85.21	0.803
BCI IV-2a	w/o TF	×	Gating	85.02	0.801
BCI IV-2a	w/o Gating	√	Conv.	82.33	0.764
BCI IV-2a	Our method	√	Gating	86.96	0.826
Self EEG-MI	Baseline	×	Conv.	86.21	0.816
Self EEG-MI	w/o TF	×	Gating	84.21	0.789
Self EEG-MI	w/o Gating	√	Conv.	83.01	0.773
Self EEG-MI	Our method	√	Gating	88.73	0.849

**Note:** TF denotes the time–frequency branch; Conv. denotes the original convolutional encoder in EEG-TransNet; Gating denotes the proposed adaptive multi-branch EEG feature gating module; w/o denotes “without”; √ and × indicate whether the corresponding module is included.

## Data Availability

The public BCI IV-2a dataset used in this study is publicly available through public channels. The self-collected EEG-MI and eye-movement datasets are not publicly available because they contain private physiological and behavioral information from participants and are subject to ethical and privacy restrictions. De-identified data or controlled-access materials may be available from the corresponding author upon reasonable academic request, subject to institutional approval, ethical compliance, and a necessary data-use agreement. The complete source code is not publicly released at this stage because the model, robotic-arm control program, and data-processing pipeline are still being optimized and integrated. Key implementation details have been added to the revised manuscript to improve methodological transparency.
